# Epstein Barr Virus: Development of Vaccines and Immune Cell Therapy for EBV-Associated Diseases

**DOI:** 10.3389/fimmu.2021.734471

**Published:** 2021-10-08

**Authors:** Xinle Cui, Clifford M. Snapper

**Affiliations:** ^1^ Genitourinary Malignancies Branch, Center for Cancer Research, National Cancer Institute, National Institutes of Health, Bethesda, MD, United States; ^2^ The Institute for Vaccine Research and Department of Pathology, Uniformed Services University of the Health Sciences, Bethesda, MD, United States; ^3^ Citranvi Biosciences LLC, Chapel Hill, NC, United States

**Keywords:** Epstein-Barr virus, EBV-associated cancer, EBV prophylactic vaccine, therapeutic EBV vaccine, adoptive T-cell therapy, chimeric antigen receptor T cell therapy, T cell receptor engineered T cell therapy

## Abstract

Epstein-Barr virus (EBV) is the first human tumor virus discovered and is strongly implicated in the etiology of multiple lymphoid and epithelial cancers. Each year EBV associated cancers account for over 200,000 new cases of cancer and cause 150,000 deaths world-wide. EBV is also the primary cause of infectious mononucleosis, and up to 70% of adolescents and young adults in developed countries suffer from infectious mononucleosis. In addition, EBV has been shown to play a critical role in the pathogenesis of multiple sclerosis. An EBV prophylactic vaccine that induces neutralizing antibodies holds great promise for prevention of EBV associated diseases. EBV envelope proteins including gH/gL, gB and gp350 play key roles in EBV entry and infection of target cells, and neutralizing antibodies elicited by each of these proteins have shown to prevent EBV infection of target cells and markedly decrease EBV titers in the peripheral blood of humanized mice challenged with lethal dose EBV. Recent studies demonstrated that immunization with the combination of gH/gL, gB and/or gp350 induced markedly increased synergistic EBV neutralizing activity compared to immunization with individual proteins. As previous clinical trials focused on gp350 alone were partially successful, the inclusion of gH/gL and gB in a vaccine formulation with gp350 represents a promising approach of EBV prophylactic vaccine development. Therapeutic EBV vaccines have also been tested clinically with encouraging results. Immunization with various vaccine platforms expressing the EBV latent proteins EBNA1, LMP1, and/or LMP2 promoted specific CD4+ and CD8+ cytotoxic responses with anti-tumor activity. The addition of EBV envelope proteins gH/gL, gB and gp350 has the potential to increase the efficacy of a therapeutic EBV vaccine. The immune system plays a critical role in the control of tumors, and immune cell therapy has emerged as a promising treatment of cancers. Adoptive T-cell therapy has been successfully used in the prevention and treatment of post-transplant lymphoproliferative disorder. Chimeric antigen receptor T cell therapy and T cell receptor engineered T cell therapy targeting EBV latent proteins LMP1, LMP2 and/or EBNA1 have been in development, with the goal to increase the specificity and efficacy of treatment of EBV associated cancers.

## Introduction

Epstein-Barr virus (EBV) is a gamma human herpesvirus that primarily infects B cells and epithelial cells. EBV is the primary cause of infectious mononucleosis (1, 2). There are ~125,000 new cases of infectious mononucleosis each year in the United States, and it is the most common cause of lost time for new Army recruits (3–5). Infectious mononucleosis could cause persistent fatigue for up to 6 months and cause severe neurologic, hematologic, or liver complications (2, 3, 6, 7). EBV is also the first human tumor virus discovered, and it has been strongly implicated in the etiology of multiple lymphoid and epithelial cancers, such as Burkitt lymphoma (BL), Hodgkin lymphoma (HL), post-transplant lymphoproliferative disorder (PTLD), nasopharyngeal carcinoma (NPC), and gastric carcinoma (GC) (8–11). Overall EBV associated cancers account for over 200,000 new cases of cancer and cause 150,000 deaths world-wide each year (3, 8, 10). Patients undergoing solid organ or stem cell transplantation are at risk of developing uncontrolled B cell proliferation due to EBV reactivation, termed PTLD that can evolve into Hodgkin lymphoma or non-Hodgkin lymphoma, and a similar phenomenon also occurs in patients with AIDS (3, 12–14). A role for EBV has also been suggested in the pathogenesis of T and NK cell lymphomas, aggressive NK cell leukemia, and lymphoepithelioma-like carcinoma of the lung, salivary gland and thymus (15–17). Many studies further suggest a possible role for EBV in the pathogenesis of several autoimmune diseases, including multiple sclerosis, systemic lupus erythematosus, rheumatoid arthritis and Sjogren’s syndrome (7, 18). In addition, there is evidence suggesting that childhood EBV infection in sub-Saharan Africa may worsen the clinical course of malaria (19). It has also been proposed that EBV infection in the oral cavity may play an important role in promoting chronic periodontitis (20).

EBV prophylactic vaccine aiming at prevention of primary EBV infection has been in development for more than 30 years, and clinical trials of therapeutic EBV vaccines targeting EBV associated cancers have been conducted for more than 10 years. Though neither an EBV prophylactic vaccine nor a therapeutic EBV vaccine has been licensed, promising progresses have been made. Encouraged by the spectacular results of CAR-T cell therapy targeting B cell antigens, CAR-T cell therapy and TCR engineered T cell therapy targeting EBV antigens are in development, with the goal of development of highly efficient treatment for EBV associated cancers and avoid the adverse effects of targeting B cell antigen.

## Epstein-Barr Virus

**Figure 1:**
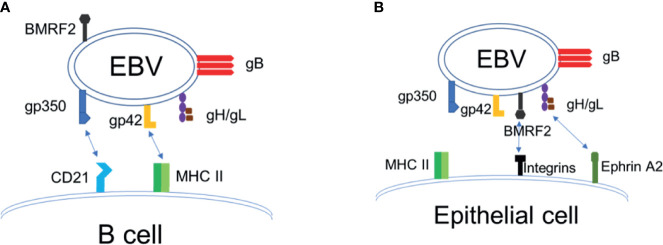
EBV entry and infection of target cells. **(A)** EBV infection of B cells. **(B)** EBV infection of epithelial cells.

EBV has a linear, double-stranded DNA genome that is approximately 170 kilobase pairs in length, which encodes more than 80 proteins and 46 functional small untranslated RNAs (21, 22). EBV is typically transmitted *via* saliva and contracted during infancy in developing countries, whereas in the developed world, it is typically contracted during adolescence (5, 23). EBV infects >95% of the world population by adulthood. The target cells of EBV are B lymphocytes and epithelial cells, and the mechanism by which EBV enters into host cells is shared in many aspects by other members of the herpesvirus family (24–28). Infection of B cells with EBV is initiated by binding of the EBV envelope protein gp350 to the complement receptor 2 (CR2)/CD21. Upon binding to B cell CR2, EBV gp42 interacts with the host cell surface MHC-II, leading to its association with the heterodimeric protein gH/gL. EBV gH/gL then activates the EBV fusion protein gB, that directly mediates viral-host cell endosomal membrane fusion [(25–27), **Figure 1A**]. EBV infection of epithelial cells involves EBV BMRF2 binding to integrins, followed by gH/gL binding to integrins and ephrin receptor A2, triggering activation of gB and fusion of the viral envelope to the plasma membrane of the epithelial cell [(7, 28–36), **Figure 1B**]. Thus, EBV envelope glycoproteins gH/gL, gB and gp350 play key roles in EBV infection of target cells, where gH/gL and gB constitute the “core fusion machinery” mediating fusion with the cell membrane (24, 25, 28). The native conformation of EBV gB is a trimer, and EBV gH and gL naturally form a heterodimer (24). EBV envelope proteins gH/gL and gB are essential for EBV infection of both B cells and epithelial cells, whereas gp350 is important for efficient infection of B cells (24, 25, 28, 33).

Once infecting the host, EBV establishes two alternative modes of infection: lytic and latent. During the lytic infection, EBV expresses more than 80 lytic proteins and these proteins facilitate the generation of new EBV viral particles and engage in immune evasion (37, 38). During the process of developing latency, EBV progresses through three different EBV latency programs characterized by a set of gradually restricted viral gene expression patterns, but no production of EBV virions. Through this process EBV develops eventual lifetime persistence in memory B cells (39, 40). Latent EBV infections play a key role in the pathogenesis of EBV associated cancer. In type III latency, all eight EBV latent antigens are expressed, including six EBV nuclear antigens (EBNA 1, EBNA2, EBNA3A, EBNA3B, EBNA 3C and EBNA 6/LP), and two latent membrane proteins (LMP1 and LMP2) (21). Latency III is mainly seen in PTLD and immunoblastic lymphomas in HIV infected patients (41). Latency II exists in HL, NPC and GC, where EBNA1, LMP1 and LMP2 are expressed (42). In type I latency, only EBNA1 is expressed and is seen in BL (21).

## EBV Infection and Infectious Mononucleosis

EBV is found in saliva, and is transmitted through kissing, coughing, intimate intact or sharing food and eating utensils (43, 44). After successful transmission, EBV infects oral epithelial cells, most likely squamous epithelial cells in the tonsil (45). EBV also infects B cells in the lymphatic tissues of Waldeyer’s Ring including the tonsils (44). It is unknown whether epithelial cells or B cells are infected first. EBV replication occurs exclusively in epithelial cells which are thus responsible for EBV transmission. Epithelial cells lack MHC-II so are unable to sequester gp42 upon release of EBV virions. These virions expressing gp42, then demonstrate a preference for infection of B cells, which express MHC-II. Likewise, the presence of gp42 blocks gH binding to integrins on epithelial cells thus inhibiting epithelial cell infection. When released from B cells, MHC-II sequesters gp42 thus producing EBV virions lacking gp42 and demonstrating a preference for infecting epithelial cells (25). In developing countries primary EBV infection usually occurs during infancy and childhood with most individuals infected by 4 years of age. In contrast, in developed countries, primary EBV infection typically occurs during adolescence or early adulthood. These differences are most likely caused by socioeconomic factors (44).

Primary EBV infection in young children usually produces no significant symptoms, whereas primary EBV infection in adolescents and young adults could cause infectious mononucleosis (IM), and up to 70% of adolescents and young adults present with the classical symptoms of IM after EBV infection (44, 46). IM is characterized by two to four weeks of fever, pharyngitis, cervical lymphadenopathy and fatigue accompanied by a massive expansion of the number of EBV-specific CD8+ T cells after an incubation period of about six weeks (44, 47). Disease severity and duration of IM correlate with CD8+ T cell counts rather than with the viral kinetics (48–50). This indicates that IM is caused by an overreaction of the immune system due to a failure in early control of viral replications, leading to an exaggerated CD8+ T cell response with consequent inflammatory cytokine release (39, 48, 51). It has been suggested that infants and children, but not older individuals produce an NK cell subset important for early EBV control, thus preventing the occurrence of IM in this population (52). The vast majority of IM cases are self-limiting with an excellent prognosis, with rare cases of severe acute complications such as splenic rupture, hepatitis and airway obstruction due to tonsil enlargement (43, 44, 53). Late complications to IM include Hodgkin lymphoma and multiple sclerosis (21). Life-long latent EBV infection establishes following primary infection (41).

## EBV Associated Cancers

EBV is the first human tumor virus identified. EBV not only causes infectious mononucleosis, it is also strongly associated with epithelial cell cancers such as nasopharyngeal cancer (NPC), gastric cancer as well as lymphoid cancers such as Burkitt lymphoma (BL), Hodgkin lymphoma (HL) and post transplantation lymphoproliferative disorder (PTLD) (8–14). NPC is endemic in southeast Asia, and the vast majority are the nonkeratinized type, accounting for 80,000 new cases each year worldwide (10, 41, 54). Nonkeratinized NPC displays a lymphoepithelial-like (LEL) appearance with a marked lymphocytic infiltration, which is 100% EBV positive (41). About 10% of gastric cancers are associated with EBV infection, have a similar LEL pathological change and are EBV positive, accounting for about 83,000 new cases each year (41, 55, 56). Essentially all Burkitt lymphoma in equatorial Africa and in Papua New Guinea, are EBV genome-positive, accounting for 7,000 new cases each year (57–59). Hodgkin lymphoma is also strongly associated with EBV, especially the mixed cellularity subtype, of which 80-90% are EBV positive (60–63). PTLD is another example that EBV plays a critical role in cancer pathogenesis, all the cases of PTLD are EBV positive, and adoptive transfer of EBV specific T cells could prevent or cure the disease (64–68). The role of EBV in cancer pathogenesis has also been confirmed in animal models, as inoculation of cotton top tamarins or humanized mice with high titers of EBV results in the development of B-cell lymphomas and lymphoproliferative disease that are seen in humans (69–78).

### Burkitt’s Lymphoma

Burkitt’s lymphoma (BL) is highly aggressive and it is the most common pediatric cancer in the world (42, 58). Based on clinical observations and disease epidemiology, BL is classified into three different forms: endemic, sporadic and immunodeficiency-associated BLs (79). Endemic BL occurs in equatorial Africa and in Papua New Guinea, where essentially all cases are EBV genome-positive (41, 80). Sporadic BL has a wide global distribution, but has a much lower frequency with only 10–15% linked to EBV except for North East Brazil where the frequency of EBV in sporadic BL exceeds 80% (81, 82). Immunodeficiency-associated BL has been diagnosed in HIV carriers who develop the lymphoma before progressing to AIDS, with an incidence of 10 to 100-fold higher than that of the sporadic BL, and about 30-40% of Immunodeficiency-associated BL are positive for EBV (41, 83, 84).

BLs have a characteristic histology with sheets of monomorphic tumor cells resembling germinal centroblasts interspersed with macrophages, and have hypermutated Ig gene sequences typical of their geminal center (GC) origin and all carry chromosomal translocations that bring the c-myc gene under the control of either the Ig heavy chain or light chain loci (84). The resultant deregulation leading to constitutive expression of c-myc proteins at high levels in BL cells, and causes uncontrolled cell growth in BL (85). The expression of EBV genes in EBV positive BL is restricted to the type I latency program, where only EBNA-1 is expressed (86). Though the mechanism of EBV in the pathogenesis of BL is still unclear, it has been shown the expression of EBNA-1 in BL cell lines promotes cell proliferation by inhibiting apoptosis (87). Also, coinfection with other pathogens seem to play an important role, such as coinfection of EBV and Plasmodium falciparum in endemic BL, and coinfection of EBV and HIV in immunodeficiency-associated BLs (88, 89).

### Hodgkin Lymphoma

Hodgkin lymphoma (HL) originates from B cell and is characterized by the presence of few malignant multinucleated giant Reed Sternberg cells surrounded by a massively outnumbered non-neoplastic inflammatory infiltrate (41). Based on the nature of the infiltrate, classical HL is divided into four histologic subtypes namely the mixed cellularity (MC), nodular sclerosing (NS) and the rarer lymphocyte rich (LR) and lymphocyte-depleted (LD) subtypes (60). A minor, non-classical subtype of HL is referred to as lymphocyte predominant (LP). About 96% of MC cases are EBV genome-positive, whereas only 15-20% of cases of NS are associated with EBV. The rarer LR subtype is associated with EBV in 40% of cases, and most LD cases are EBV+ (42, 90). EBV is not associated with the non-classical LP subtype (91). Overall, about 30-40% of HL cases in North America and Europe were reported to be EBV positive, but in Latin America, Africa and Asia, EBV was found in almost 100% of all HL cases (91).

The Reed Sternberg cells in HL are B cells arrested at the germinal center (GC) or post-GC stages of B cell differentiation where somatic mutations in immunoglobulin (Ig) V genes are detected (92–95). The Reed Sternberg cells of the classical type of HL fail to express most B cell-specific genes, including Ig genes (91). Though the role of EBV in HL pathogenesis is still not fully understood, it is reported that all EBV-positive tumors consistently exhibit Latency II infection with high levels of the LMP1 and LMP2A proteins maintained in every Reed-Sternberg cell (41). LMP1 functions as a constitutively active variant of the CD40 receptor, and activates the aberrant transcriptional programs in Reed Sternberg cells, including the NF-κB, JAK/STAT, AP-1, and (PI3K)/AKT pathways (96–103). LMP1 could also promote the survival of EBV-infected B cell receptor (BCR)-negative Reed Sternberg progenitors and increase the expression of anti-apoptotic molecules, including BCL2 and MCL1 (104–106). LMP2A mimics BCR, allows B cell development in the absence of BCR signaling, activates RAS/PI3K/AKT signaling and the mTOR pathway (107–111). LMP2A is also critical for the EBV-induced immortalization of BCR-negative GC B cells and LMP2A expression in different B cells systems can induce many of the cellular transcriptional changes characteristic of Reed Sternberg cells (112–117).

### Post-Transplant Lymphoproliferative Disorder

Post-transplant lymphoproliferative disorder (PTLD) is an uncontrolled B cell proliferation observed in some patients after solid organ or hematopoietic stem cell transplantation that can lead to non-Hodgkin and Hodgkin lymphoma (118). The prevalence of EBV-associated PTLD ranges from 1-20%, with incidence varying according to the type of allograft, age, and pretransplant EBV serostatus of transplant recipient (42). PTLD occurs as a result of increased proliferation of B cells due to either primary EBV infection, or reactivation of EBV from latently infected cells after treatment with immunosuppressant to avoid allograft rejection (119). The genome of EBV has been found in all of the B cells from PTLD patients. The use of immune suppressive drugs after organ transplant leads to depletion of EBV-specific T cells, and the impairment of EBV-specific T cell mediated immune surveillance results in uncontrolled lymphoproliferative blast, which causes PTLD in transplant recipients (119–122). PTLD display Latency III infection, and all the eight EBV latent antigens are expressed, including six EBV nuclear antigens EBNA 1, EBNA2, EBNA3A, EBNA3B, EBNA 3C, EBNA 6/LP and two latent membrane proteins LMP1 and LMP2 (41, 42). These latent EBV proteins play key roles in the uncontrolled proliferation of B cells, and they are also the targets of EBV specific CD8+ T cells (21, 123).

### Nasopharyngeal Cancer

Nasopharyngeal cancer (NPC) is a squamous cell epithelial tumor that arises from the lateral wall of nasopharynx, including the fossa of Rosenmüller and superior posterior wall (124). NPC shows remarkable variation in ethnic and geographical distribution, with about 80% cases reported in Southern China and Southeast Asia (42, 125). NPC rates are low in the United States and Europe, the tumor occurs at intermediate to high incidence throughout South-East Asia and reaches its peak in populations of Southern Chinese decent where NPC is endemic, with a rate that is 30-fold higher than that of the United States and Europe (126). NPC is classified into two histological variants, namely squamous cell carcinomas (SCCs) and undifferentiated carcinomas of the nasopharyngeal type (UCNT) (42, 127). In a non-endemic region, about 63% of NPC cases are UCNT, while in Southern China, more than 95% of the cases are UCNT (128). Irrespective of geography and of incidence rate, all cases of undifferentiated NPC worldwide are EBV-associated, and the EBV genome present in every malignant cell (10).

Though genetic element and lifestyle/environmental factor contribute to NPC risk, EBV infection plays a critical role in NPC pathogenesis (41, 42). The viral infection in NPC epithelial cells is clonal, developed from clonal proliferation of single EBV infected epithelial cell (42, 129). EBV viral gene expression in NPC tumor cells is an intermediate form, Latency I/II, all tumors express EBNA1, the non-coding EBERs and BART-miRs, and LMP2, whereas LMP-1 has been found in about two-thirds of NPC cases (86, 130). The BART-miRs and LMP2 promote epithelial cell growth (131–134). LMP1 activates the NFkB signaling pathway, which is a consistent feature of all NPCs (135).

### Gastric Cancer

Gastric cancer (GC) has a worldwide annual incidence of over 950,000 cases, and ranks as the third leading cause of cancer-related mortality globally (42). Though 75% of gastric cancers appear to be linked to H. pylori infection, up to 10% of gastric cancers are EBV-positive, accounting for 90,000 new cases each year (55, 56). The EBV associated gastric cancers appear to form a clinically and pathogenetically distinct subgroup, occurs predominantly in the proximal stomach including the cardia, fundus and body, tend to present earlier in life, have a lower rate of lymph node involvement and a relatively favorable prognosis (56, 136–138). EBV associated gastric cancer is classified into three histological subtypes, lymphoepithelioma like carcinoma (LELC)-type, conventional adenocarcinoma (CA)-type, and carcinoma Crohn’s, and more than 90% of EBV associated gastric cancers show LELC-type morphology (138). Latency I or intermediate Latency I/II EBV infection are detected in EBV associated gastric cancer cells, and all the malignant cells within an individual tumor carry the same monoclonal virus genome (139). Though the role of EBV in the pathogenesis of gastric carcinoma is poorly understood, EBV-positive gastric cancer shows a distinctive hyper-methylated genome (140, 141). It is proposed that EBV actively drives oncogenic change through epigenetic modification of the host cell genome, and silences tumor suppressor genes such as p16 and E cadherin (142).

## EBV and Multiple Sclerosis

Multiple sclerosis (MS) is the most common autoimmune demyelinating disease, affecting both the brain and spinal cord. It is a lifetime, potentially debilitating condition with both remitting/relapsing and progressive phases. It typically occurs in young adults, especially Caucasians with a prevalence of 1/1,000 in this latter population and affects ~2.5 million people worldwide (143). EBV is recognized as the strongest infectious risk factor for this disease. Essentially all MS patients are EBV seropositive, and although still controversial, EBV may be a necessary pre-requisite for MS development (144). Several lines of evidence suggest an etiological role of EBV in MS, including but not limited to a high titer of anti-EBNA1 antibodies observed several years before MS onset, preliminary success of EBV-specific T cell therapy for treating progressive MS, and intriguing data from animal modeling (145–148). Of note, individuals carrying the HLA-DR15 gene (found in ~25% of the overall population) who develop IM have at least a 10-fold increased risk of developing MS (149). MS typically develops >5 years after IM. Although a prophylactic EBV vaccine holds promise for markedly reducing the incidence of MS, confirmation will likely require long-term follow-up of individuals receiving the vaccine for other indications, most likely prevention of IM.

## Prophylactic EBV Vaccine

An EBV prophylactic vaccine holds great promise for prevention of cancers caused by EBV infection, as has been the case for prophylactic vaccines against human papilloma virus (HPV) and hepatitis B virus that cause ~600,000 and ~400,000 cases of new cancers each year respectively (10, 150). An EBV prophylactic vaccine would also be the most cost-effective approach for the management of infectious mononucleosis as well as EBV associated autoimmune disease such as MS (**Table 1**). A prophylactic EBV vaccine uses the strategies to induce antibody response mainly neutralizing antibodies to block EBV infection of its target cells, whereas non-neutralizing antibodies as well as cell mediated immune response further improve prophylactic efficacy. The target cells of EBV are mainly B cells and epithelial cells, and EBV requires multiple envelope proteins for cell entry. EBV infection of B cells requires envelope proteins gp350, gH, gL, gB and gp42, whereas EBV infection of epithelial cells requires envelope proteins BMFR2, gH, gL and gB, therefore these envelope proteins could be excellent EBV prophylactic vaccine candidates [(7, 25–27, 33), **Table 1**].

**Table 1:** Summary of prophylactic EBV vaccines.

Platform/Antigen/Adjuvant	Animal/ Clinical trial	Published year	Results
**1. EBV envelope protein vaccines**			
Subunit vaccine, purified full length gp340 from virus, with liposome, Freund’s adjuvant, lipid A	Mice,	1984	Antibody responses were induced (70).
	Cottontop		
	tamarins		
Subunit vaccine, Purified full length gp340 from virus	Cottontop	1985	Protection against malignant lymphoma (151).
	tamarins		
Subunit vaccine, purified gp350/gp220 from yeast and mammalian cells	Rabbits	1988	EBV-specific neutralizing antibodies were induced (152).
Subunit vaccine, purified gp340, incorporated into immune-stimulating complexes	Cottontop	1988	Protection against malignant lymphoma (153).
	tamarins		
Subunit vaccine, recombinant gp340 adjuvanted with Alum	Cottontop	1994	Protection of 3/5 cottontop tamarins against malignant lymphoma (154).
	tamarins		
Subunit vaccine, recombinant single chain gp350 with Freund’s adjuvant versus Alum	Rabbits	1999	High titers of neutralizing antibody elicited (155).
Subunit vaccine, recombinant single chain gp350 with AS04 versus alum	Phase II	2007	Induced neutralizing antibodies in 70% of human subjects, and decreased IM by 78% (156).
Subunit vaccine, recombinant tetrameric versus monomeric gp350^1-470^ adjuvanted with alum/CpG-ODN	Mice	2013	Tetrameric gp350 induced ∼20- fold higher titers of IgG and >19-fold higher neutralizing titers at the highest dose (157).
Subunit vaccine, self- assembling nanoparticles expressing gp350^1-123^	Mice	2015	gp350-nanoparticle elicited 10- to 100-fold higher neutralizing titers compared to soluble gp350 (158).
Subunit vaccine, recombinant monomeric gH/gL, trimeric gH/gL and trimeric gB, adjuvanted with alum/CpG-ODN	Rabbits	2016	Trimeric and monomeric gH/gL, trimeric gB, and tetrameric gp350 induced EBV-neutralizing titers >100-, 20-, 18-, and 4-fold higher, respectively, than monomeric gp350 (159).
Subunit vaccine, Fc-fused gp350 dimer with Alum	Mice	2018	Elicited significantly higher neutralizing titers than gp350 monomer (160).
Subunit vaccine, self-assembling nanoparticles expressing gH/gL or gH/gL/gp42 with SAS adjuvant	Mice, cynomolgus macaques	2019	gH/gL and gH/gL/gp42-ferritin nanoparticles elicited >40- and ~4-fold higher neutralization titers for B cells in monkeys compared with soluble proteins; for epithelial cells, >25- and ~4-fold higher neutralizing titers were elicited. (161).
Subunit vaccine, recombinant trimeric gB, monomeric gH/gL and their combination, adjuvanted with alum/CpG-ODN	Rabbits, humanized mice	2021	Sera from rabbits immunized trimeric gB or monomeric gH/gL protected humanized mice from lethal dose EBV challenge and markedly decreased EBV loads. Immunization with the combination of gB and gH/gL elicited strong synergistic neutralizing activity (162).
**2. EBV Virus-Like particle (VLP) vaccines**			
EBV-derived VLP, produced via the deletion of the EBV terminal repeats, EBNA2, EBNA 3A, 3B and 3C, LMP1 and BZLF1	Mice	2011	EBV-specific humoral and cellular immune responses were induced (163).
EBV-derived VLP, produced by fusing EBNA1 and EBNA3C to the major tegument protein BNRF1	Humanized mice	2018	Potent CD4+ T cell responses elicited, and EBV loads were reduced (164).
Newcastle disease virus (NDV) VLP, expressing gp350/220 Ectodomain	Mice	2015	Elicited neutralizing antibody responses, but not higher than that of soluble gp350/220 (165).
NDV VLP, expressing gH/gL-EBNA1 or gB-LMP2	Mice	2017	Elicited EBV-specific T-cell responses and higher EBV neutralizing titers (166).
**3. Synthesized mRNA EBV vaccines**			
Synthesized mRNA encoding gp350, gB, gH/gL and gp42	Mice	2021	Elicited ~20- and ~100-fold higher neutralizing activities for B cells and epithelial cells respectively compared to human sera.

### Recombinant EBV Envelope Protein Vaccines

Early efforts in EBV vaccine development were focused on gp350 (**Table 1**). EBV gp350 is the most abundant EBV envelope protein, and about half of the EBV neutralizing activity in EBV seropositive human sera is against gp350 (7, 161). Purified or recombinant gp350 was shown to protect cotton top tamarins from lymphoma caused by EBV infection, and similar results were reported with adenovirus or vaccinia virus expressing gp350 (69–76). Phase I and II studies of a recombinant gp350 produced in Chinese hamster ovary cells showed that the recombinant gp350 induced neutralizing antibodies in humans in 70% of the subjects, and reduced the rate of infectious mononucleosis by 78% in the vaccinated subjects but did not prevent EBV infection (156, 167). This is most likely because gp350 is not strictly essential for EBV virus infection of B cells but only important for efficient infection, as well as the inability of gp350 induced antibodies to protect against EBV infection of epithelial cells (24, 25, 28, 33, 168).

Our group was the first to report in 2016 that recombinant EBV gH/gL and gB proteins induced markedly higher EBV neutralizing antibodies compared to gp350, where a trimeric form of gH/gL and a tetrameric form of gp350 elicited significantly higher EBV neutralizing activities compared to their monomeric counterparts (159). This was confirmed by the recently published study by Bu et al. that EBV gH/gL or gH/gL/gp42 nanoparticles induced potent neutralizing antibody responses in mice and non-human primates, which blocked EBV-target cell fusion and prevented EBV infection of B cells and epithelial cells (161). Though these nanoparticle EBV vaccine candidates induced 10- to 1000-fold higher titers of neutralizing antibodies compared to that of soluble proteins, as the gH, gL and/or gp42 proteins were highly packed into the nanoparticles, the expression of native conformational epitopes of these EBV envelope proteins could be prevented (161). It was reported that virus like particles and nanoparticles could induce quantitatively high antibody responses whereas recombinant proteins expressing native epitopes could elicit antibody responses that are high both quantitatively and qualitatively (169–172). This has been confirmed with the herpes virus recombinant envelope protein vaccine candidates produced in our laboratory including EBV gH/gL and EBV trimeric gB (159, 173, 174).

EBV gH/gL and gB constitute the core fusion machinery, which play the critical roles for EBV fusion and entry into all target cells, thus making the inclusion of EBV gH/gL and gB more promising as prophylactic EBV vaccine candidates than the use of gp350 alone (24, 25, 28). As EBV gH/gL and gB could both elicit neutralizing antibodies, the presence of gH/gL neutralizing antibodies and gB neutralizing antibodies simultaneously would most likely exhibit synergistic effects (7, 24, 33). In this regard, we recently demonstrated that mixing EBV gH/gL anti-sera with EBV gB anti-sera resulted in strong synergistic neutralizing activity for both B cells and epithelial cells. This may have been due to the ability of gH/gL and gB antibodies to block different steps in EBV infection of target cells (162). Immunization with the combination of EBV gH/gL and trimeric gB also elicited markedly higher EBV neutralizing activities for both B cells and epithelial cells as compared to that induced by immunization with EBV gH/gL or trimeric gB alone, demonstrating strong synergistic effects of EBV core fusion envelope proteins in elicitation of neutralizing antibodies (162). The strong synergistic effects are most likely due to the sequential coordination of these envelope proteins in mediating EBV entry and infection of target cells, suggesting that the combination of EBV core fusion machinery envelope proteins gH/gL and trimeric gB could be a highly effective EBV prophylactic vaccine. Recombinant gp350 also demonstrated synergistic EBV neutralizing activities when used with EBV gH/gL and/or gB (175). Further, we recently reported that the immune sera from rabbits immunized with EBV gH/gL or trimeric gB protected humanized mice from death after lethal dose EBV challenge and markedly decreased the EBV in peripheral blood (162). Collectively, these data strongly suggest that the combination of EBV gH/gL, gB and gp350 could be an ideal EBV prophylactic vaccine that can elicit markedly high synergistic EBV neutralizing activity with the potential to induce sterilizing immunity. EBV gp350, gH, gL, and gB can also induce CD4+ and CD8+ T cell immune responses, and have demonstrated to inhibit proliferation of EBV-infected primary B cells *in vitro* before latency was established (40, 176, 177). The T cell immune responses induced by EBV gp350, gH, gL, and gB could further increase the efficacy of a prophylactic vaccine by promoting the killing of recently infected epithelial cells and B cells and stopping B-cell transformation if the EBV is not blocked by neutralizing antibodies.

### Virus-Like Particle EBV Vaccines

Virus-Like particle (VLP) vaccines have been successfully developed for hepatitis B virus and human papilloma virus, but the development of VLP vaccines for EBV has met challenges. The first successful EBV VLPs were created by the deletion of the EBV terminal repeats, and potential oncogenes namely EBNA2, 3A, 3B and 3C, LMP1 and BZLF1 (163). These EBV VLPs were shown to elicit EBV-specific humoral and cellular immune responses after immunization in mice (163). Later, the viral packaging and nuclear egress proteins BFLF1/BFRF1A were further deleted to improve safety, and the EBV VLPs produced could induce comparable CD4+ T cell responses as that of wildtype EBV, but the responses against structural proteins were reduced. More immunogenic EBV VLPs were made by fusing latent antigens EBNA1 and EBNA3C to the major tegument protein BNRF1 of EBV. The EBV VLPs produced were able to stimulate potent CD4+ T cell responses against structural as well as latent EBV protein epitopes, and reduced EBV loads in the peripheral blood of humanized mice after immunization (164). However, the possibility of repacking of EBV DNA remains a safety concern for the EBV-derived VLPs. Another approach used a novel Newcastle disease virus (NDV) VLP platform displaying the EBV gp350/220 ectodomain, but the EBV neutralizing titers elicited in mice were not significantly higher than that induced by soluble gp350/220 (165). The NDV VLP platform was subsequently used to produce gH/gL-EBNA1 VLPs and gB/LMP2 VLPs. Both elicited higher EBV neutralizing titers in mice, but not comparable to that elicited by UV-inactivated EBV, suggesting potency concerns with the NDV VLP EBV vaccines (166).

### Synthesized mRNA EBV Vaccines

The rapid and successful development of synthesized mRNA vaccines against SARS-CoV2 for the COVID-19 pandemic has encouraged the development of synthesized mRNA EBV vaccines (178, 179). After demonstrating its mRNA vaccine candidate encoding gp350, gB, gH/gL and gp42 induced significantly higher EBV neutralizing titers in mice compared to human sera, Moderna has initiated phase I clinical trial of its mRNA EBV vaccine. However, there are potential challenges with this approach. First, whether mRNA vaccines could induce long-term memory responses in humans remains to be determined, and may require repeated booster immunizations to maintain high titers of neutralizing antibodies and potent T cell immune responses. This would be unacceptable for pathogens such as EBV that rarely cause a medical emergency. Second, mRNA is an intrinsic adjuvant that may limit multi-component formulations because of unacceptable side effects. Currently it has been reported that mRNA vaccines against SARS-CoV2 may have caused myocarditis and pericarditis in a small number of subjects. Long-term adverse effects as well as adverse effects after multiple repeated immunizations are still unknown.

### Viral Vector EBV Vaccines

The viral vector vaccine platform has mainly been used for the development of therapeutic EBV vaccines, which are discussed in detail later in the therapeutic EBV vaccine section.

## Therapeutic EBV Vaccine

The immune system plays a critical role in the control of tumors, and the immune-based tumor-specific therapeutic approaches could be highly effective with limited adverse effects (21). Therapeutic EBV vaccines aim at boosting the existing or inducing novel antiviral adaptive immune responses in patients with EBV-associated cancers (21). The targets of EBV therapeutic vaccines are focused on EBNA1, LMP2 and/or LMP1, as these proteins are involved in the modulation of key factors that contribute to the transformation of normal cells into tumors [(180–185), **Table 2**]. Most therapeutic EBV vaccination approaches have focused on patients with NPC, and early clinical trials were done with dendritic cells (DCs)-based EBV vaccines (21). Autologous monocyte-derived dendritic cells (DCs) from NPC patients pulsed with HLA-A11-, -A24-, or -B4-restricted LMP2 epitopes boosted EBV-specific CD8+ T-cell responses in nine of 16 patients, and induced partial tumor regression in two patients (191). In another phase I clinical trial where 16 HLA-A2+ NPC patients were vaccinated with autologous DCs pulsed with HLA-A2-restricted LMP2A peptides, the LMP2-specific T-cell response was improved in 9 of 16 patients, which correlated with a modest decrease in serum EBV DNA levels (187).

**Table 2:** Summary of therapeutic EBV vaccines.

Platform/Antigen	Disease	Clinical trial	Published year	Results
**1. Dendritic cells (DCs)-based EBV vaccines**				
Autologous monocyte-derived DCs pulsed with LMP2 peptides	NPC	Phase I	2002	EBV-specific CD8+ T-cell responses boosted in 9/16 patients, and partial tumor regression induced in 2/16 patients (186).
Autologous DCs pulsed with LMP2A peptides	NPC	Phase I	2013	LMP2-specific T-cell response improved in 9/16 patients, with decreased serum EBV load (187).
**2. Recombinant viral vector vaccines**				
Adenoviral vector expressing LMP2	NPC	Phase I	2016	LMP2-specific CD3+ CD4+ cells increased (188).
Modified vaccinia ankara (MVA) expressing EBNA1 and LMP2 as a fusion protein (MVA-EL)	NPC	Phase I	2013	Two-fold increase in the T-cell response to EBNA1 and/or LMP2 in 15/18 patients treated (189).
MVA-EL	NPC	Phase I		Increased numbers and differentiationof CD4+ and CD8+ T-cells to EBNA1 and LMP2 in 4/8 patients (190).

Recombinant viral vector vaccines were later developed to present a wide range of epitopes to improve efficacy. One of the approaches used a recombinant adenoviral vector to deliver LMP2 antigen, and demonstrated a dose dependent increase in the proportion of LMP2-specific CD3+ CD4+ cells in the peripheral blood of immunized NPC patients in a clinical trial (188). A modified vaccinia ankara (MVA) expressing the carboxyl terminus of EBNA1 and full-length LMP2 as a fusion protein (MVA-EL) was shown to efficiently expand the EBNA1- and LMP2-specific CD4+ and CD8+ T cells from the peripheral blood lymphocytes of seropositive healthy donors *in vitro* (192). A phase I clinical trial of the MVA-EL vaccine carried out in Hong Kong where NPC patients in remission received three intradermal MVA-EL immunizations at 3-weekly intervals, and demonstrated a two-fold increase in the T-cell response to one or both vaccine antigens in 15 of 18 treated patients (189). Further, these T-cell responses were mapped to known CD4+ and CD8+ T-cell epitopes of EBNA1 and/or LMP2 (189). A second phase I clinical trial of the MVA-EL vaccine conducted in UK showed an increase in CD4+ and CD8+ T-cell responses to one or both of the antigens in 8 of the 14 NPC patients tested, as well as increased differentiation and functional diversification in the EBNA1- and LMP2-specific CD4+ and CD8+ cells (190).

Though the EBV therapeutic vaccine clinical trials have shown many potential clinical benefits, further remains to be done. The DC vaccines can only deliver limited antigen epitopes and are expensive to prepare (21). Recombinant viral vector vaccines can deliver a wide range of epitopes, but the anti-viral vector immune responses elicited after repeated immunizations are a big obstacle, which could markedly decrease the efficacy of viral vector vaccines. Different vaccine platforms should be explored for the development of EBV therapeutic vaccines. Although T-cell responses were believed to be critical in controlling EBV-associated cancers, high levels of neutralizing antibodies are associated with a lower risk of NPC, and NPC progression has been shown to correlate with active EBV replication and high titers of EBV in the peripheral blood, thus a vaccine inducing potent EBV neutralizing antibodies may reduce the risk of EBV-related cancers (21, 193). The role of the neutralizing antibody response in EBV-associated malignancies needs to be explored further. A vaccine platform delivering EBNA1, LMP1/LMP2, gp350, gH/gL and gB simultaneously may significantly increase the efficacy of a therapeutic vaccine against EBV associated cancers.

## Immunotherapy Against EBV Associated Cancer

### Adoptive T-Cell Therapy

Adoptive T-cell therapy (ACT) has been successfully used for the treatment of PTLD, and the reconstitution of cellular immunity provides a powerful mechanism to control EBV-associated PTLD [(21), **Table 3**]. The first ACT clinical trial demonstrated that infusion of donor-derived EBV-specific T lymphocytes into allogeneic hematopoietic stem cell transplantation (HSCT) patients with donor-derived EBV-associated immunoblastic lymphoma induced a complete regression in 5/5 patients, but graft-versus-host disease (GvHD) was developed due to the alloreactive T cells (194). Later EBV-specific cytotoxic T lymphocytes (CTLs) were obtained by *in vitro* stimulation with EBV-transformed B lymphoblastoid cell lines (LCLs), synthetic peptides or recombinant viral vectors, and followed by selection with TCR tetramer binding or IFN-γ capture. Clinical trials demonstrated that these *in vitro*-expanded donor-derived EBV-specific CTLs could be used effectively for the prevention and treatment of PTLD in HSCT patients with minimal alloreactivity (64–66). Similar strategies were also used to expand *in vitro* the autologous EBV-specific CTLs from highly immunosuppressed solid organ transplantation (SOT) patients and successfully controlled EBV-associated PTLD (195–197). Encouraged by the excellent results of treatment of PTLD with ACT, ACTs have been developed for NPC and HL using *in vitro* expanded CTLs targeting type II latency antigens EBNA1, LMP1 and LMP2 [(198, 199, 206–208), **Table 3**]. Clinical trials with these expanded CTLs significantly increased response rates as well as overall survival in both NPC and HL patients (186, 200, 201).

### EBV Specific T Cell Receptor Engineered T Cell Therapy

T cell receptor (TCR) engineered T cell therapy is a promising approach to cancer treatment, and has been used in the treatment of HPV associated cancer targeting E7 antigen with impressive results (209–211). TCRs that recognize EBNA3A, EBNA3B, LMP1, LMP2, BRLF1 and BMLF1 have been generated from specific CD8+ T cell clones, and tested their effectiveness in eliminating EBV transformed B cells (212–214). It was reported that the T cells transduced with these T cell receptors recognized autologous LCLs weakly, partially due to limited latent EBV antigen expression in LCLs. However, the tumor progression of a NPC cell line expressing LMP2 implanted into nude mice was significantly attenuated by LMP2 TCR transduced T cells (215). It was hypothesized that some of the transduced TCR α and β chains might pair with the endogenous TCR β and α chains of the T cells, which reduce the number of transduced TCR and thereby prevented efficient LCL recognition (216). To improve correct pairing of transduced TCR α and β chains, chimeric TCRs were generated, where the variable domains of TCR α and β chains from EBV specific T cell clones were fused to the corresponding mouse TCR constant regions (217). The stability of chimeric TCRs was further improved by introducing an additional disulfide bond between the α and β chain of the murine TCR constant regions (202, 218). As a result, T cells transduced with a chimeric TCR recognizing LMP2 were able to kill up to half of co-incubated LMP2 positive cells *in vitro* and to suppress LMP2 expressing tumor cell growth in immune compromised mice [(202), **Table 3**]. Similar results were observed with a TCR recognizing EBV LMP1, and T cells transduced with LMP1 specific TCR doubled the survival of immune compromised mice challenged with LMP1 expressing tumor cells (203).

**Table 3:** Summary of EBV immune cell therapies.

Platform	Disease, clinical trial/Animal model		Published year	Results
**1. Adoptive T-cell therapy**				
Donor-derived EBV-specific T lymphocytes	PTLD	I	1994	Complete regression in 5/5 patients, but graft-versus-host disease developed (194).
EBV-specific cytotoxic T-lymphocyte (CTL) lines from donor	PTLD, HSCT	I	1995	Complete regression of immunoblastic lymphoma in 1/1 patients, EBV reactivation controlled in 3/3 patients without lymphoma (64).
Donor-derived polyclonal CD4+ and CD8+ T-cell lines	PTLD, HSCT	I	1998	Complete regression of immunoblastic lymphoma in 2/2 patients, EBV reactivation controlled in 6/6 patients without lymphoma (65).
Autologous EBV-specific CTL lines	PTLD, SOT	I	1999	Significant regression of the PTLD in 1/1 patient (195).
Autologous EBV-specific CD8 and CD4 lymphocytes	PTLD, SOT	I	2006	Complete regression of liver PTLD in 1/1 patient, prevention of PTLD in 12/12 patients (196).
Autologous EBV-specificcytotoxic T lymphocytes	SOT	I	2002	Decrease EBV load, prevention of PTLD in 7/7 patients (197).
Autologous EBV-specific cytotoxic T lymphocytes	NPC	I	2005	Complete response in 4/10 patients and partial response in 2/10 patients (198).
Autologous EBV-specific cytotoxic T lymphocytes	NPC	I/II	2010	Complete response in 7/15 patients and partial response in 3/15 patients (199).
Autologous EBV-specific cytotoxic T lymphocytes	NPC	II	2014	Complete response in 3/35 patients and partial response 22/35 patients (200).
Autologous EBV-specific cytotoxic T lymphocytes targeting LMP2	Lymphoma	II	2014	Complete response in 11/21 patients and partial response 2/21 patients (201).
**2. EBV Specific T Cell Receptor (TCR) engineered T cell therapy**				
Autologous CD8 and CD4 Lymphocytes expressing LMP2 specific TCR	NSG mouse		2015	Lysed LMP2+ NPC cells and inhibited tumor growth in a mouse model (202).
	NPC model			
Autologous CD8 and CD4 Lymphocytes expressing LMP1 specific TCR	NSG mouse tumor model		2018	Doubled the survival time of mice bearing tumor (203).
**3. EBV Specific Chimeric Antigen Receptor (CAR) T cell therapy**				
CD8 and CD4 Lymphocytes Expressing LMP1 specific CAR	NSG mouse		2014	Killed 70% of LMP1 overexpressing NPC cells in vitro, and significantly reduced the growth of NPC tumor overexpressing LMP1 (204, 205).
	tumor model		2019	

### EBV Specific Chimeric Antigen Receptor T Cell Therapy

T cells expressing chimeric antigen receptors (CARs) targeting CD19, CD20, CD22 and CD30 were able to kill around 50% of different B cell lymphomas in cell culture, and have provided spectacular results in clinical trials, with 60% of B cell lymphoma and leukemia patients in complete remission after treatment (219–223). However, significant adverse effects of cytokine release syndrome and neurotoxicity have also been observed due to the abundance of target cells, including both normal and malignant B cells after infusion of CAR-T cells (123). Further, deficiencies in humoral immune responses could be induced due to the persistent non-specific killing of B cells and predispose the patients for respiratory and gastrointestinal infections (224). To avoid the adverse effects of B cell antigen specific CAR-Ts, EBV specific CAR-Ts have been explored for treatment of NPC. LMP1 specific CAR-T cells could kill up to 70% of LMP1 overexpressing NPC cells *in vitro*, and intratumoral injection of anti-LMP1 CAR-T cells significantly reduced the growth of NPC tumor overexpressing LMP1 in immune compromised mice [(204, 205), **Table 3**]. Though it is questionable whether the LMP1 specific CAR-T cells could target the tumor cells in NPC patients where LMP1 expression is usually much lower, the results are encouraging. Clinical trials using LMP1 specific CAR-T cells for the treatment of NPC are underway, but no results have yet reported. EBV LMP2 is another promising target for treatment of EBV associated cancers.

## Conclusion

EBV infects more than 95% of the human population, causes IM in 70% of adolescents and young adults in developed countries, accounts for 1.5% of all cancers worldwide and represent 1.8% of all cancer deaths. An EBV prophylactic vaccine holds great promise for prevention of EBV associated cancers such as BL, HL, PTLD, NPC, and GC, and would also be the most cost-effective approach for the management of IM as well as EBV associated autoimmune disease such as MS. Though VLP, viral vector and mRNA platforms have been used for the development of an EBV prophylactic vaccine, the approach using recombinant EBV envelope proteins has made advancement the most. EBV envelope proteins including gH/gL, gB, gp350 play key roles in EBV entry and infection of its target cells, and neutralizing antibodies elicited by each of these proteins have shown to prevent EBV infection of target cells, markedly decrease EBV titers in the blood of humanized mice, and prevent their death after challenge with lethal dose EBV. Recent studies demonstrated that immunization with the combination of recombinant EBV gH/gL and gB proteins markedly increased the EBV neutralizing activity compared to immunization with individual protein. These data suggest that the combination of EBV gH/gL, gB and gp350 could be an ideal EBV prophylactic vaccine that can elicit markedly high EBV neutralizing activity with the potential to induce sterilizing immunization, prevent primary EBV infection and therefore prevent EBV associated cancers as well IM and MS.

For patients with EBV-associated cancers, therapeutic EBV vaccines targeting latent proteins EBNA1, LMP2 and/or LMP1 are promising treatments. Though the current DC-based and viral vector EBV therapeutic vaccines have shown significant limitations, different vaccine platforms targeting both latent and lytic envelope proteins including EBNA1, LMP1/LMP2, gp350, gH/gL and gB simultaneously can be explored to improve efficacy. Immune cell therapies for EBV-associated cancers have emerged as another highly promising treatment. ACT has demonstrated to cure and prevent PTLD, and has shown to be highly effective in the treatment of NPC and HL by targeting latent proteins EBNA1, LMP1/LMP2. To further improve efficacy and minimize adverse effects, EBV specific CAR-T and TCR engineered T cell therapies targeting EBV latent protein LMP1, LMP2 and/or EBNA1 have been developed, with the goal of specific killing of EBV+ cancer cells highly efficiently.

## Author Contributions

XC prepared the manuscript draft, CMS provided comments to the text and figures, read and approved the final manuscript. All authors contributed to the article and approved the submitted version.

## Funding

This research was supported in part by the Intramural Research Program of the National Cancer Institute, National Institutes of Health. This research was also supported by the Dean’s Research and Education Endowment Fund from Uniformed Services University of the Health Sciences (USUHS). USUHS had no involvement in design of study, data analysis or interpretation, nor manuscript preparation or study publication.

## Author Disclaimer

The opinions expressed herein are those of the authors and are not necessarily representative of those of USUHS, the United States Army, Navy or Air Force, or the Department of Defense (DOD). The funders had no role in the design of the study; in the collection, analyses, or interpretation of data; in the writing of the manuscript, or in the decision to publish the results.

## Conflict of Interest

CMS was employed by Citranvi Biosciences LLC. XC and CMS are inventors of a patent for vaccine development using herpesvirus trimeric gB proteins, and a pending patent using key envelope proteins combination in vaccine development.

## Publisher’s Note

All claims expressed in this article are solely those of the authors and do not necessarily represent those of their affiliated organizations, or those of the publisher, the editors and the reviewers. Any product that may be evaluated in this article, or claim that may be made by its manufacturer, is not guaranteed or endorsed by the publisher.
